# Trou maculaire post traumatique chez un enfant de six ans: documentation par OCT

**DOI:** 10.11604/pamj.2014.18.90.3724

**Published:** 2014-05-26

**Authors:** Chama Daoudi, Mina Laghmari

**Affiliations:** 1 Université Mohammed V Souissi, Service d'Ophtalmologie A de l'hôpital des spécialités, Centre hospitalier universitaire, Rabat, Maroc

**Keywords:** Trou maculaire, OCT, rétinographie, macular hole, OCT, Retinography

## Image en médecine

Les trous maculaires post-traumatiques sont, chez l'enfant, la première cause de trou maculaire, contrairement à celles de l'adulte. La tomographie en cohérence optique permet désormais un suivi plus objectif de ces déchirures rétiniennes. Nous rapportons le cas d'un enfant de 6 ans victime d'un traumatisme contusif de l'oeil droit avec cataracte post traumatique, l'examen du fond d'oeil a montré un trou fovéolaire d'environ un tiers du diamètre papillaire avec des plis rétiniens de part et d'autre de la lésion. L'OCT montrait un trou fovéolaire de pleine épaisseur. L'acuité visuelle était limitée à compte les doigts de près. Une surveillance régulière durant 6 mois et appuyée par l'OCT à objectiver la persistance du trou maculaire. Les trous maculaire secondaires sont majoritairement causés par des traumatismes du globe oculaire de cinétique violente. Dans ce cas, l'origine du trou maculaire post-contusif est liée à la déformation brutale du globe oculaire, lors de la compression antéro-postérieure avec étirement de la rétine. Ces forces de traction rétiniennes transversales entraînent la rupture de la macula (zone de fragilité du pôle postérieur rétinien). Il existe une proportion des trous maculaires post traumatiques qui cicatrisent spontanément, la petite taille du trou (200 µm) favorise ces fermetures spontanées, ce qui n'est pas le cas de notre patient.

**Figure 1:**
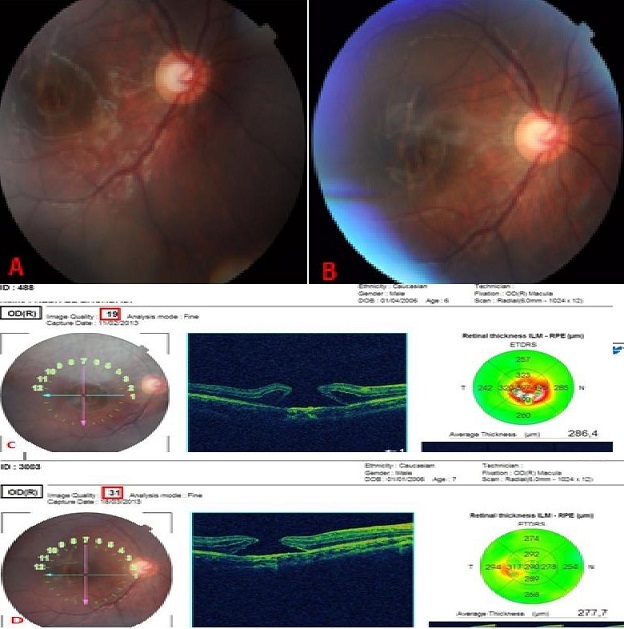
A) Rétinographie couleur: trou maculaire de pleine épaisseur, pli rétinien transversal J2; B) Cliché OCT-3 à J2: trou maculaire de pleine épaisseur post traumatique; C) Rétinographie couleur: trou maculaire de pleine épaisseur après 6 mois; D) Cliché OCT-3 après 6 mois: trou maculaire de pleine épaisseur post traumatique

